# Filamentation initiated by Cas2 and its association with the acquisition process in cells

**DOI:** 10.1038/s41368-019-0063-0

**Published:** 2019-10-03

**Authors:** Lei Wang, Xin Yu, Mengjie Li, Guiqin Sun, Lin Zou, Tiansheng Li, Linlin Hou, Yameng Guo, Danfeng Shen, Di Qu, Xunjia Cheng, Li Chen

**Affiliations:** 1 Department of Medical Microbiology, Key Laboratory of Medical Molecular Virology of Ministries of Education and Health, School of Basic Medical Sciences, Fudan University, 131 Dongan Road, Shanghai, China; 2 College of Medical Technology, Zhejiang Chinese Medical University, Hangzhou, China

**Keywords:** Cellular microbiology, CRISPR-Cas systems, CRISPR-Cas systems

## Abstract

Cas1-and-Cas2-mediated new spacer acquisition is an essential process for bacterial adaptive immunity. The process is critical for the ecology of the oral microflora and oral health. Although molecular mechanisms for spacer acquisition are known, it has never been established if this process is associated with the morphological changes of bacteria. In this study, we demonstrated a novel Cas2-induced filamentation phenotype in *E. coli* that was regulated by co-expression of the Cas1 protein. A 30 amino acid motif at the carboxyl terminus of Cas2 is necessary for this function. By imaging analysis, we provided evidence to argue that Cas-induced filamentation is a step coupled with new spacer acquisition during which filaments are characterised by polyploidy with asymmetric cell division. This work may open new opportunities to investigate the adaptive immune response and microbial balance for oral health.

## Introduction

The clustered regularly interspaced short palindromic repeats (CRISPR)-associated protein (CRISPR-Cas) adaptive immune system is widespread in bacteria and archaea.^1–4^ The system was detected in a panel of oral pathogens, including the major oral pathogens *Streptococcus* mutants^5,6^ for caries, *Porphyromonas gingivalis*^7^ for periodontal disease, and *Enterococcus faecalis*^8,9^ for endodontis. The CRISPR-Cas system may play a critical role in microflora and oral health. Recent studies have indicated that the diversity of the CRISPR system(s) of the oral microflora plays an important role in the salivary microflora and may serve as a potential clinical index for oral health.^10–12^ The CRISPR-Cas system was associated with the pathogenic potential of endodontic oral microbes as well as the status of drug resistance.^8,9^

The main role of the CRISPR-Cas system is to maintain the fidelity of the genome by removing invading phages and other episomal DNA elements in a two-step process known as new spacer acquisition and spacer-guided immune execution. New spacer acquisition, also known as genomic recording, is an essential and necessary process for CRISPR-Cas system-mediated bacterial adaptive immunity.^13^ In acquisition, fragmented DNAs derived from chromosome and episomal DNAs, such as phages, plasmids and synthetic oligonucleotides, form a protospacer and integrate at the first repeat of the CRISPR array by a sequential cutting and fixing reaction.^14–17^ Transcription of this new spacer produces a target-specific CrRNA (CRISPR RNA) for guided target elimination.^18^ Adaptive immunity is achieved through acquisition and the following selections.^19^ In addition to its classical physiological function to eliminate phages, the CRISPR-Cas system is also implicated in drug resistance,^20^ pathogenesis,^21^ and synthetic biology.^22^ However, the roles of the system and individual Cas proteins in other cellular functions remain largely unknown.

Early studies have demonstrated that both the Cas1 and Cas2 proteins are core elements for acquisition. Qimron’s group first demonstrated that co-expression of the Cas1 and Cas2 proteins (Cas1-Cas2) was sufficient for new spacer acquisition in *E. coli* BL21.^14^ This original observation was further confirmed by several other groups.^15,16,23^ Biochemical and structural analyses have shown that Cas1 and Cas2 could form a complex to mediate new spacer acquisition.^15,16,23^ More recently, genomic recording was achieved by introducing a synthetic protospacer that co-expressed Cas1 and Cas2 into *E. coli* BL21.^24^

Despite all this progress, the impacts of the Cas1-and-Cas2-mediated new spacer acquisition process on bacterial cells are largely unknown. In this paper, we report the role of Cas2 in generating a novel filamentation phenotype and demonstrate a novel filamentation phenotype coupled with new spacer formation.

## Results

### Cas2-induced filamentation and its regulation by Cas1

**Fig. 1:**
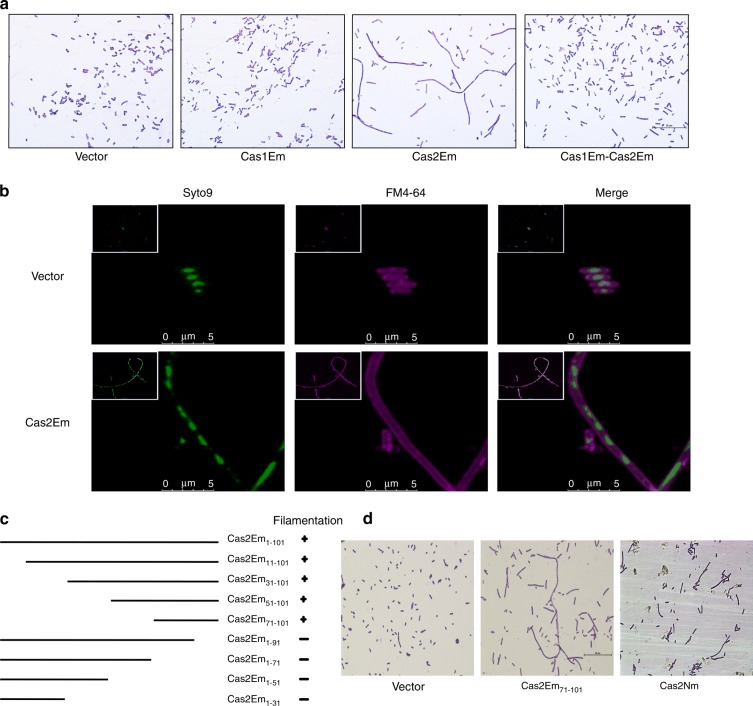
The C-terminal 30 amino acids are necessary and sufficient for Cas2Em-initiated filamentation in BL21(DE3). **a** Representative images of BL21(DE3) cells with expression of the vector control, Cas1Em, Cas2Em and Cas1Em-Cas2Em (scale bar, 25 μm). **b** Fluorescent images of Cas2Em-initiated filamentation labelled by SYTO 9 (green) and FM 4–64 (magenta) to indicate the DNA and membrane, respectively (scale bar, 5 μm). **c** The presence (+) and absence (-) of the filamentation phenotype with Cas2Em constructs, including four N-terminal and four C-terminal deletions. **d** Representative images of BL21(DE3) cells with expression of the vector control, Cas2Em_71-101_ and Cas2Nm

**Fig. 2:**
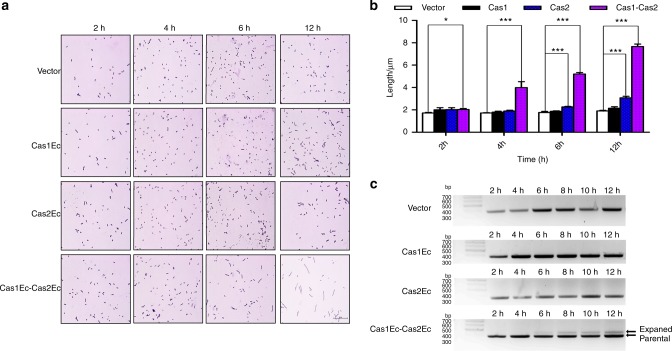
Cas1Ec-Cas2Ec-initiated filamentation in BL21-AI. **a** Representative images of BL21-AI cells with the vector control, Cas1Ec, Cas2Ec and Cas1Ec-Cas2Ec at 2 h, 4 h, 6 h and 12 h after induction (scale bar, 25 μm). **b** Quantitative analysis of the length (*n* = 4 per group, mean with S.D.) of bacterial cells. Significant differences between the control and treatment groups are indicated by *P < 0.05 and ***P < 0.000 1. **c** The results of the in vivo new spacer acquisition assay for the tested cells. The parental and expanded bands are indicated. New spacer acquisition is defined by the appearance of the expanded band. Representative data from at least three independent experiments are presented

In previous studies, a multidrug-resistant clinical strain of *Elizabethkingia meningoseptica* (*E. meningoseptica*, FMS-007) was isolated from a non-Hodgkin lymphoma patient and subjected to genomic and functional genome analysis.^25–27^ In the standard complete genome of FMS-007 (no. CP006576.1), a type II-C CRISPR-Cas adaptive immune system with only three Cas proteins (Cas9, Cas1 and Cas2) was identified (Fig. S1). In an attempt to study the role of *E. meningoseptica* Cas proteins (CasEm), we expressed CasEm in *E. coli* BL21. To our surprise, a significant Cas2Em-initiated filamentous phenotype was observed (Fig. 1a). The phenotype was Cas2Em specific and was downregulated by co-expression of Cas1Em (Fig. 1a). Furthermore, confocal analysis of the cells fluorescently by SYTO 9 for the nucleoid and FM 4–64 for the membrane of live bacteria showed that filaments contained multiple nucleoids within a whole and continuous membrane (Fig. 1b). This Cas2-initiated filamentation phenotype was further confirmed by the induced expression of the Cas2 proteins from another pathogen, *Neisseria meningitidis* (Cas2Nm) (Fig. 1d), and *E. coli* (Cas2Ec) (Fig. 2a, b). In addition, a motif of 30 amino acids at the carboxyl-terminal end of Cas2Em was identified as the functional domain for Cas2Em-initiated filamentation (Fig. 1c, d).

Although Cas2Ec-induced filaments were relatively shorter than those induced by the Cas2Em protein, the degree of filamentation was enhanced by co-expression of Cas1Ec (Fig. 2a, b). Since co-expression of Cas1-Cas2 can mediate filamentation and new spacer acquisition (Fig. 2), the potential relationship(s) between filamentation and new spacer formation were further explored by a panel of assays.

### Filamentation occurred prior to new spacer acquisition

**Fig. 3:**
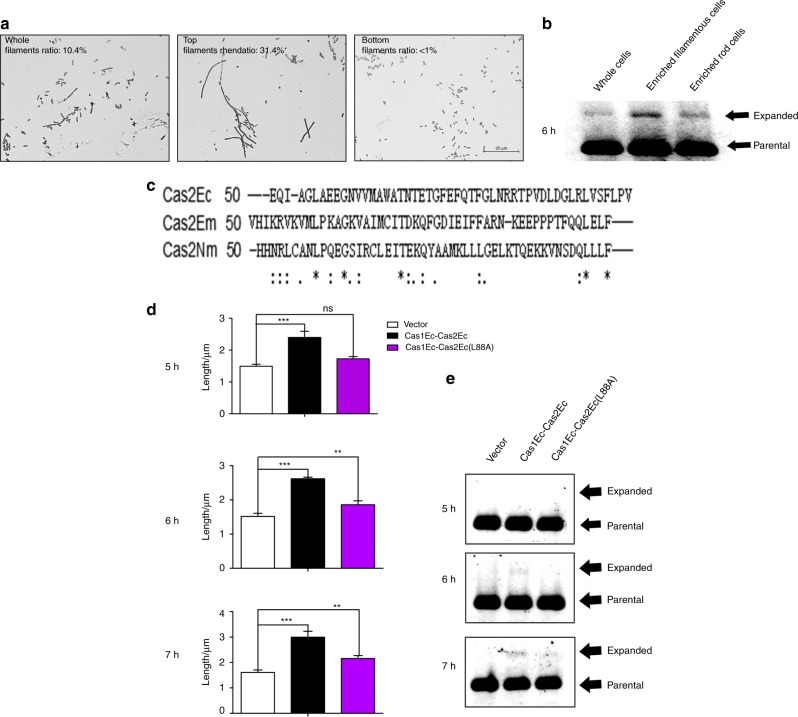
Cas1Ec-Cas2Ec-initiated filamentation is associated with new spacer acquisition. **a** The filamentation phenotypes after the separation of filaments and rod cells by the filter-based method: Top: cells retained by the filter; Bottom: cells filtered through the membrane (scale bar, 25 μm). **b** New spacer acquisition of unfiltered cell and filter-separated fractions at 6 h after induction of Cas1Ec-Cas2Ec expression. **c** Sequence comparison of Cas2 proteins from *E. coli*, *E. meningoseptica* and *N. meningitidis* generated by Clustal Omega. Identical amino acids are indicated by “*”; similar amino acids in the same group are indicated by “:”; and amino acids from different groups are indicated by “.”. **d** Quantitative analysis of the average length (*n* = 4 per group, mean with S.D.) of cells induced by Cas1Ec-Cas2Ec and/or the Cas2Ec mutant in BL21-AI compared with that induced by the vector control. Differences between the groups are indicated by “ns”: not significant; **P < 0.001 and ***P < 0.000 1. **e** The results of an in vivo new spacer acquisition assay for Cas2Ec mutants. In both **b** and **e**, the parental and expanded bands are indicated by arrows. Representative data from at least three independent experiments are presented

A dynamic filamentation/acquisition assay was established to determine the temporal sequence of Cas1-Cas2-mediated filamentation and new spacer formation. After IPTG-induced expression of Cas1Ec-Cas2Ec, filamentation was first detected at approximately 3 h, and new spacer formation appeared at ~6 h (Fig. 3a). This result clearly indicated that filamentation is an event that occurred before new spacer acquisition.

### Acquisition of spacers is common in filamentous cells

In addition, a filter-based method was adapted to separate filamentous and rod-shaped cells in the new spacer formation assay.^28^ Filamentous cells were significantly enriched in the fraction retained by the filter (31.4%), while the rod-shaped cells were dominant (>99%) in the fraction through the filter (Fig. 3a). The same amounts of the DNAs purified from the unfiltered sample and two filtered fractions were analysed for new spacer formation. The acquisition of new spacers was significantly higher in the filamentous-enriched fraction than in the non-enriched fraction at early time points (Fig. 3b). Semi-quantitative analysis of the substrate and product bands in each fraction indicated that the acquisition reaction was significantly enriched in the filaments.

### Mutations in Cas2 abrogated filamentation and spacer acquisition

To define the critical region in Cas2Ec responsible for filamentation and new spacer formation, an alignment of Cas2Ec with Cas2Em and Cas2Nm was conducted for the conserved motifs and amino acids (Fig. 3c). A panel of mutations in the conserved amino acids was generated in Cas2Ec for Cas2-initiated filamentation and in Cas1Ec-Cas2Ec for the dynamic filamentation/new spacer formation assay, respectively. The results demonstrated that Cas2Ec (L88A) was defective in Cas2Ec-initiated filamentation (Fig. S2), and the entry points for both filamentation and new spacer formation by Cas1Ec-Cas2Ec (L88A) were significantly delayed (Fig. 3d, e). The ratios of acquisition initiated by wild-type Cas1Ec-Cas2Ec and Cas1Ec-Cas2Ec (L88A) at early time points (5, 6, and 7 h) were 0.2%, 0.8%, and 5.9%, vs. 0.1%, 0.1% and 0.8%, respectively. Collectively, these results indicated that Cas2-initiated filamentation and new spacer formation in adaptive immunity are tightly associated and that Cas2-initiated filamentation is an early event for new spacer acquisition.

### Cas1Ec-Cas2Ec-initiated filamentation leads to asymmetric cell division

**Fig. 4:**
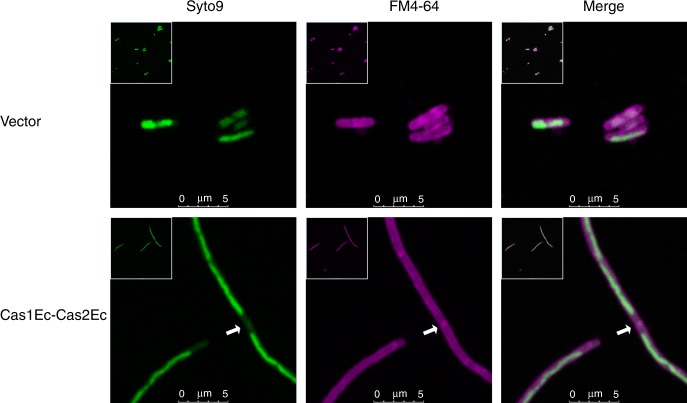
A polyploidy-like structure was detected in Cas1Ec-Cas2Ec-initiated filaments. Fluorescent images of Cas1Ec-Cas2Ec-initiated filamentation labelled by SYTO 9 (green) and FM 4–64 (red) to indicate the DNA and membrane, respectively (scale bar, 5 μm). The membrane junctions are indicated by white arrows

**Fig. 5:**
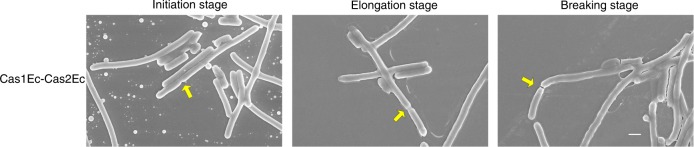
Asymmetric cell division was detected at the distal end of Cas1Ec-Cas2Ec-initiated filament. The process of asymmetric cell division detected by SEM (scale bar, 1 μm). The sites where the division would occur are indicated by yellow arrows

To further characterize Cas2-initiated filamentation, the filaments induced by Cas1Ec-Cas2Ec were subjected to live-cell immunostaining and scanning electron microscopy (SEM) analysis. Filaments with a continuous chromosome illustrated by SYTO 9 (green) staining in an extended membrane compartment with a junction at the distal end were detected (Fig. 4), consistent with a septum-like structure ~2 μm away from the distal end detected by SEM (Fig. 5), suggesting that the filaments are polyploidy and capable of asymmetric division at the end. In addition, representative images with characteristics of septum formation, elongation, and breaking were observed (Fig. 5), providing additional evidence for asymmetric division of filaments for adaptive acquisition.

## Discussion

A novel filamentation phenotype induced by Cas proteins was reported (Fig. S3). Early studies have demonstrated that bacterial filamentation can be detected under various environmental and internal stresses, such as nutrient deficiency,^29^ UV radiation,^30^ failure in cell division,^31^ antibiotic treatment^28,32^ and attacks from the host immune system.^33,34^ Clinical investigation has indicated that filamentation is an active strategy adapted by bacteria to survive in hosts.^33–36^ More importantly, Goldthwait’s group reported in 1967 that a filamentous stage was observed during prophage elimination in *E. coli* cells.^37^ In this study, we demonstrated for the first time that filamentation is an early step associated with genomic recording via the Cas2 and Cas1 proteins. A nuclease-free motif in the C-terminal domain of Cas2 was identified as the functional domain for filamentation, and more studies are required to verify the molecular and cellular function of this motif.

Although different levels of filamentation initiated by the induced expression of Cas2Em and Cas2Ec were detected in *E. coli*, both processes were regulated by the paired Cas1 protein. Cas2Ec-induced short filamentation was enhanced by Cas1Ec co-expression, while Cas2Em-induced long filamentation was decreased by Cas1Em co-expression. Because the Cas1-Cas2 complex is required for the cutting and fixing steps in new spacer formation,^15,16,24^ and DNA damage could cause filamentation,^28,32–34^ our results indicated that Cas2Ec-mediated filamentation is upregulated by DNA damage caused by the Cas1Ec-Cas2Ec complex via its recognition and cutting at the repeats in *E. coli*. Because the Cas1Em-Cas2Em complex cannot recognize the species-specific repeats in *E. coli*, the formation of the complex might interfere with the interaction between Cas2Em and the division complex (Fig. S4) for filamentation.

Filaments with a polyploidy-like structure plus a partition ring at the end were detected by imaging analysis. Polyploidy formation is a common survival and evolution mechanism adapted by eukaryotic organisms,^38–41^ such as hyphae of Candida,^42^ cysts in amoeba,^43^ and polyploidy giant cells in tumours.^44,45^ In addition to genome duplication, cells in the polyploidy stage are usually elongated and/or have enlarged shapes.^44,46,47^ Although filamentation of bacterial cells is known as a common phenotype associated with environmental stresses and pathological conditions,^47^ its active role in adaption, such as genomic recording, is under investigation. Success in the new spacer formation at the CRISPR site requires not only protospacer formation but also transient chromosomal breakage. Because both processes are vital challenges to the monoploid prokaryotic bacterial cell, the formation of a transient polyploidy-like formation could be a strategy adopted by the bacterium to achieve both tasks.

Adaptive immunity is a way for pathogens to survive in various clinical challenges, such as emerging phage therapy^48^ and drug resistance.^20^ The Cas protein-initiated morphological change reported in this paper may serve as a new target for drug development.

## Materials and methods

### Reagents, strains and plasmids

Tryptone and yeast extract for Luria Bertani (LB) medium were obtained from Oxoid. NaCl and agar were obtained from Sinopharm Chemical Reagent Co., Ltd. Antibiotics and isopropyl-β-D-thiogalactopyranoside (IPTG) were obtained from INALCO SPA MILANO. L-arabinose was obtained from LABLEAD. High-fidelity PCR polymerase and Taq PCR polymerase were obtained from TaKaRa. Fastdigest enzymes and T4 DNA ligase were from Thermo Fisher Scientific. The bacterial genomic DNA extraction kit was from TIANGEN. The bacterial strains, plasmids and oligonucleotides used in this study are listed in Supplementary table 1.

### Plasmid construction

Plasmid construction and mutagenesis were conducted as previously described.^26^ The paired oligos 4011F*/*4011R, 5011F*/*5011R and 4011F/5011R were used to clone *cas1Em, cas2Em* and *cas1Em-cas2Em* from *Elizabethkingia meningoseptica* FMS-007,^25^ respectively. The paired oligos 4021F*/*4021R, 5021F*/*5021R, and 4021F*/*5021R were used to clone *cas1Ec, cas2Ec* and *cas1Ec-cas2Ec* from *Escherichia coli* K12 W3110,^49^ respectively. The paired oligos 5041F/5041R were used to clone *cas2Nm* from a clinically isolated *Neisseria meningitidis* serogroup A strain.^50^ The deletion mutations from the N-terminal end and C-terminal end of *cas2Em* were constructed by introducing new start codons and terminal stop codons in oligos. The paired mutation oligos for L88A (*cas2Ec*) were used to construct the mutation. The paired oligos 5F11F/5F11R and 6H23F/6H23R were used to clone *cas2Em-Flag* and *ftsZ-HA*, respectively. Sequences of all clones were verified by sequencing.

In this study, two groups of Cas proteins were subjected to investigation: exogenous Cas2Em and Cas2Nm proteins and endogenous proteins, including Cas1Ec and Cas2Ec.

### New spacer acquisition assay

The assay was conducted following a standard protocol reported in previous studies,^14–16,23^ with modifications. The tested genes were transformed into *E. coli* BL21-AI (Invitrogen). Three single colonies from each tested strain were inoculated in LB medium containing 50 μg/ml kanamycin and aerated at 37 °C for 16 h. The overnight cultures were diluted 300 times in the same medium with inducing reagents (0.2% (wt/vol) L-arabinose and 0.2 mM IPTG) and aerated at 37 °C for 16 h. A total of 100 μl of culture was collected for genomic DNA extraction. The same amount of DNA purified from each sample was subjected to a specific PCR designated for the new spacer acquisition with the primers MG7F/RE10R. The PCR-amplified fragments were analysed on 2–2.5% agarose gels for new spacer acquisition.

### Dynamic filamentation/acquisition assay

Overnight cultures of the tested strains were diluted 300 times in fresh LB medium containing a selective compound (50 μg/ml kanamycin) and inducing reagents (0.2% (wt/vol) L-arabinose and 0.2 mM IPTG) and aerated at 37 °C for 16 h. Cultures were collected at different time points for genomic DNA extraction and imaging. The same amount of genomic DNA was subjected to the standard new spacer acquisition assay described above. Cells were subjected to Gram staining and imaged with a light microscope (ECLIPSE50i; Nikon).

### Measurement of cell length and statistical analysis

Statistical analyses were performed with GraphPad Prism 5.0 software. Microscopic images randomly selected from four independent experiments were analysed. The average length of 20 randomly selected cells in an image was measured first by the Neuron J plug-in in ImageJ software^51^ and then subjected to sequential statistical analysis with one-way ANOVA and Bonferroni’s multiple comparison test.

### Enrichment of filaments and rod cells

Based on a filter method,^28^ cells were separated into fractions of rod cells and filaments for the dynamic filamentation/acquisition assay. A sterile 1.2 μm syringe filter (Thermo Fisher Scientific catalogue number 41225-GM) was used for this purpose. The enriched filaments and the rod cells were collected for imaging analysis and acquisition assay.

### Confocal microscopy analysis of filaments

Cells were subjected to confocal analysis following a published protocol with modifications.^52^ For living bacterial cell staining, 100 μl cells were washed three times in PBS and loaded on silane-coated glass slides with Cytospin 4 centrifugation (Thermo Fisher Scientific). Cells were stained with SYTO 9 (Molecular Probes catalogue number T13320, diluted 600 times in PBS) for chromosomes at room temperature for 30 min, followed by one-minute staining on ice with FM 4–64 (Molecular Probes, catalogue number S34854, dissolved in 10% glycerol-PBS with 1.25 mg/ml of 1,4-diazabicyclo (2,2,2) octane) for membranes. The images of the stained samples were observed with laser scanning confocal microscopy (TCS SP8; Leica).

### SEM analysis of filaments

Cells collected at 16 h in the dynamic filamentation/acquisition assay were subjected to SEM analysis. One microlitre of cells were mixed with 1 ml of PBS and 100 μl of heparin, vortexed and centrifuged at 500 rpm for 5 min at room temperature. The supernatant was collected and centrifuged for 10 min at 2000 rpm. Bacterial cells in the pellet were fixed in 1 ml of 2.5% glutaraldehyde in PBS (pH 7.4) at 4 °C for 2 h. Following a reported protocol,^53^ the bacterial cells were washed 3 times in PBS, fixed on the slides and dehydrated by ethanol. With an HCP-2 instrument (Hitachi, Japan), the bacterial cells were critically point dried using liquid CO_2_. The samples were imaged with an FEI NOVA NANOSEM450 scanning electron microscope (Thermo Fisher Scientific).

### Immunoprecipitation assays

Cas2-Flag on pET-28a, FtsZ-HA on pET-11a and the vector controls were co-transformed into *E. coli* BL21-AI.^16^ Stable transformants selected on LB plates with kanamycin (50 μg/ml) and ampicillin (100 μg/ml) were cultured in LB medium with the two antibiotics plus 0.2% (wt/vol) L-arabinose and 0.2 mM IPTG at 37 °C for 3 h. Cells were pelleted by centrifugation at 4000 rpm for 5 min and subjected to a standard ultra-sonication protocol described in an early publication.^26^ The supernatants were collected after a 10 min centrifugation at 12,000 rpm, and the same protocol was repeated 2 times. The lysates without debris were subjected to an immunoprecipitation assay^23^ with anti-HA beads (Millipore catalogue number IP 0010) for FtsZ and anti-Flag beads (Millipore catalogue number FLAGIPT1) for Cas2. The total proteins eluted from the beads were separated on 4–12% Bis-Tris Plus gels (Invitrogen catalogue number NW04122BOX) with MES SDS running buffer (Invitrogen catalogue number NP0002); transferred onto a PVDF membrane (Invitrogen catalogue number IB24001) by iBlot II (Invitrogen); and subjected to standard western analysis with an HRP-conjugated anti-Flag antibody (Sigma-Aldrich catalogue number A8592-1MG) and HRP-conjugated anti-HA antibody (Sigma-Aldrich catalogue number H6533).

## Supplementary information

Supplementary Figures.

Supplementary table 1.
