# Supplementary figures and images for: Filamentation initiated by Cas2 and its association with the acquisition process in cells
Source: Int J Oral Sci. 2019 Oct 3;11(3):29. doi: 10.1038/s41368-019-0063-0 (PMC6802651; doi:10.1038/s41368-019-0063-0)

**Supplementary figure 1**


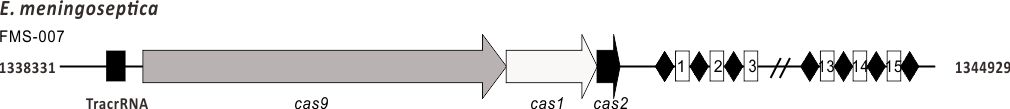


**Supplementary figure 2**


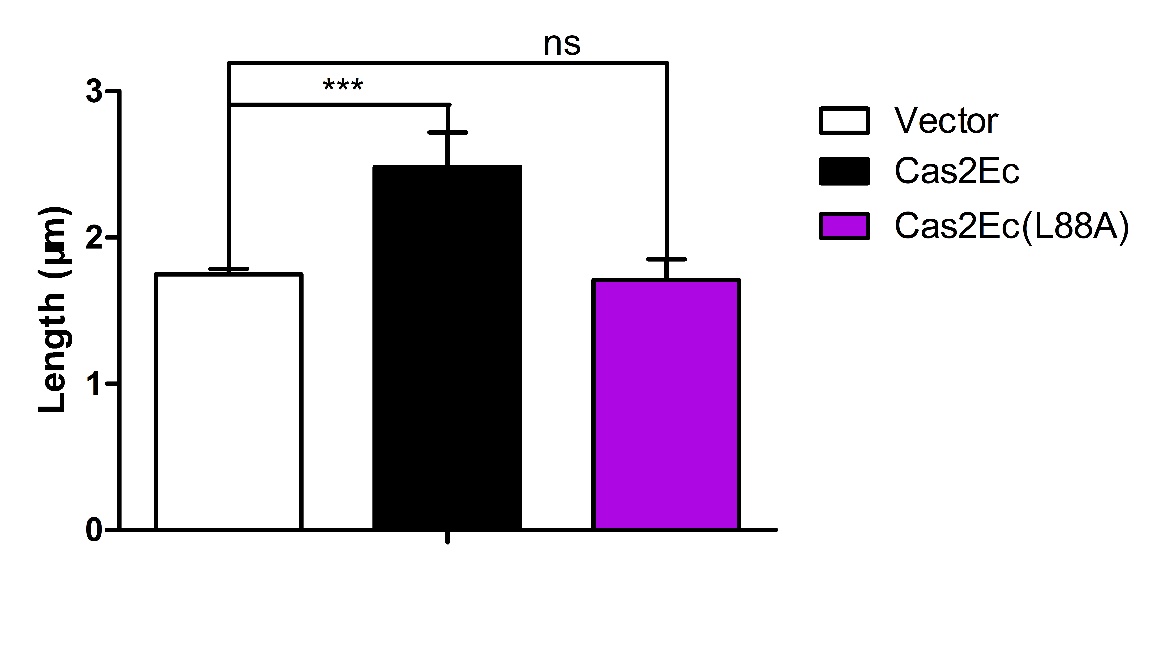


**Supplementary figure 3**


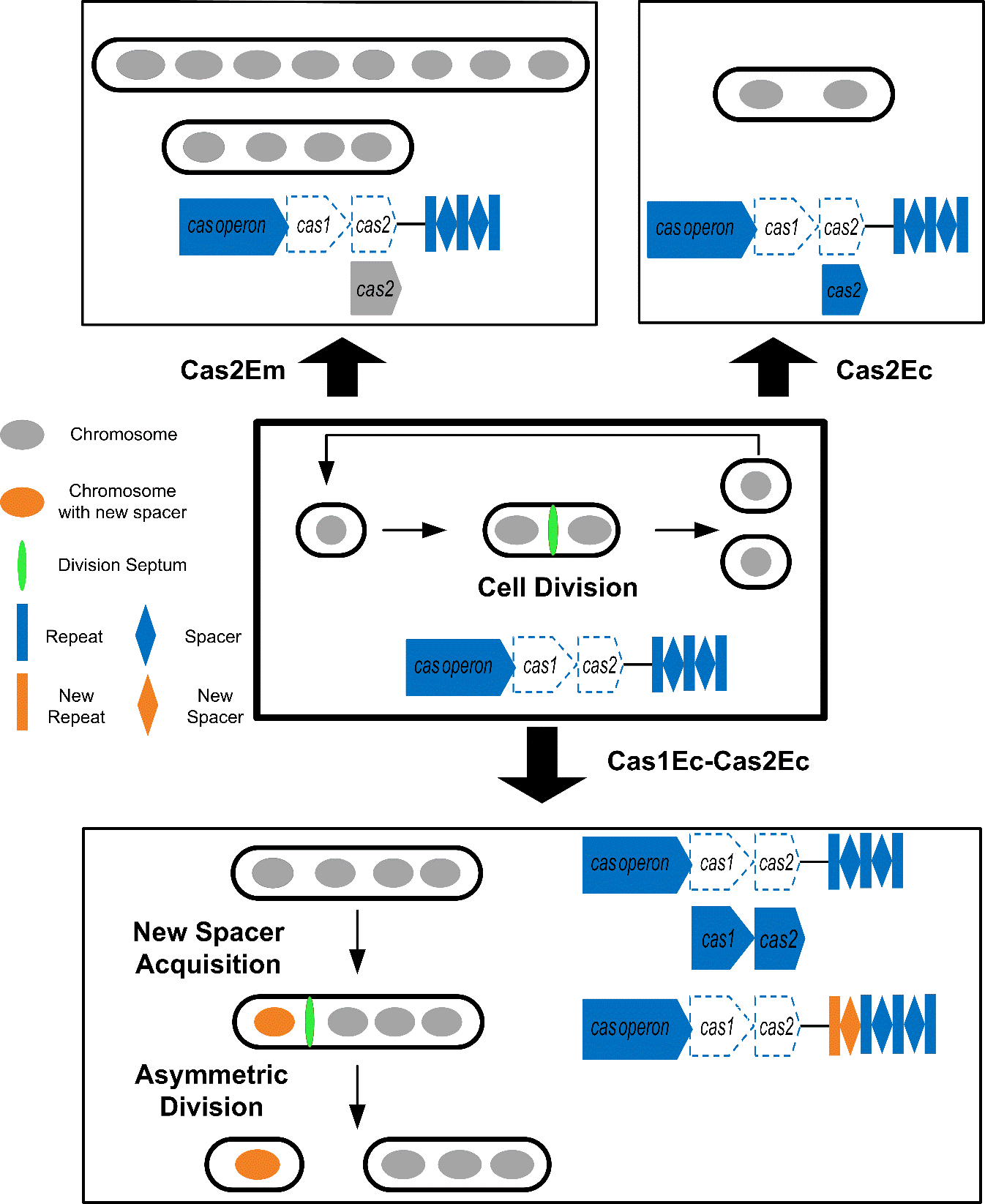


**Supplementary figure 4**


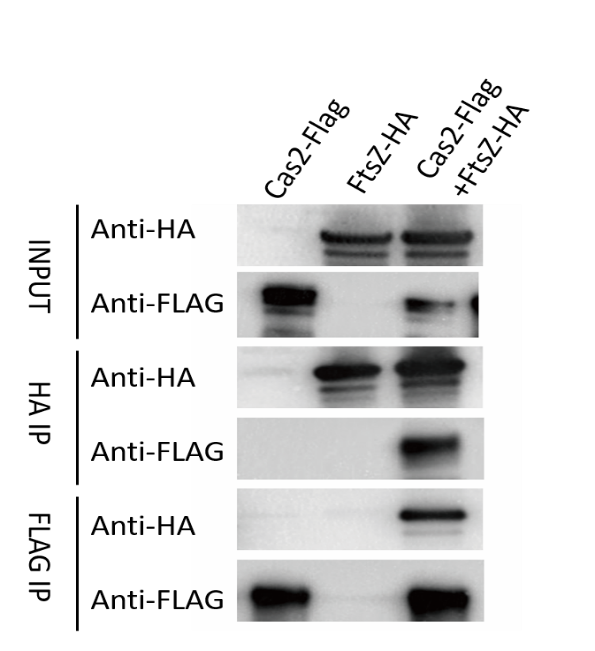

Supplement: Supplementary file 1 — Supplementary Figures. [file 41368_2019_63_MOESM1_ESM.docx]
